# Pinta tu Raya ASI [Set your Limit LIKE THIS]: an educational intervention using immersive reality to prevent Child Sexual Abuse in Mexico

**DOI:** 10.3389/fpubh.2025.1686104

**Published:** 2026-01-21

**Authors:** Leonor Rivera-Rivera, Marina Séris-Martínez, Gabriel González-Serna, Noé Alejandro Castro-Sánchez, Dolores González-Hernández, Alberto Jiménez-Tapia, Sandra Treviño-Siller

**Affiliations:** 1 Instituto Nacional de Salud Pública, Cuernavaca, Mexico; 2 Tecnológico Nacional de México / Centro Nacional de Investigación y Desarrollo Tecnológico, Cuernavaca, Mexico; 3 Instituto Nacional de Psiquiatría Ramón de la Fuente Muñiz, Mexico City, Mexico

**Keywords:** child sexual abuse, prevention, educational intervention, elementary school, virtual reality

## Abstract

**Background:**

Child Sexual Abuse (CSA) is a global social and health problem requiring a comprehensive, multi-level prevention approach. School-based education is recognized as one of the most effective prevention strategies.

**Objective:**

This project aims to test the effectiveness of the preventive intervention, “Pinta tu Raya ASI” (Set your Limit LIKE THIS), to prevent CSA. Methods: This is a two-arm matched cluster-randomized controlled trial of an educational intervention to prevent CSA in Mexican elementary school children using Immersive Virtual Reality (IVR). The study will be administered in clusters of public and private schools in urban and rural areas across two Mexican states. The intervention group will receive a learning session with an IVR animation, while the control group will receive the intervention only after the final knowledge acquisition measurement.

**Results:**

The project is expected to enhance elementary students’ knowledge of self-esteem, body safety, and rights to help them prevent child violence in Mexico.

**Ethics:**

The study was approved by the National Institute of Public Health’s Commissions of Research, Ethics, and Biosafety. Ethical safeguards include obtaining informed consent from guardians and assent from minors, providing trained staff, continuous monitoring during the IVR session, and having a specialized psychology team for case management and referral.

**Trial registration:**

The study was approved by the Commissions of Research, Ethics and Biosafety at the National Institute of Public Health: code CI: 1713 V47.

## 1 Introduction

### 1.1 Prevalence of CSA

According to the Pan American Health Organization (PAHO), Child Sexual Abuse (CSA) is defined as: “The involvement of a child or adolescent in a sexual activity that he or she does not fully understand and to which he or she is not capable of giving informed consent, or for which he or she is not developmentally ready and cannot give consent, or which violates the laws or taboos of society” (p. VII) (1). CSA is a global, social, and health concern, independent of socioeconomic status (2), and is an egregious form of violence that violates children’s rights to live free from violence (3).

Research indicates that more than 25% of children have experienced CSA before the age of 18 (4–6). Systematic reviews report rates ranging from 18 to 20% in girls and from 8 to 10% in boys globally (7), with other studies citing worldwide prevalences between 8 and 31% in girls and 3 and 17% in boys (4). In low- and lower-middle-income countries surveyed between 2007 and 2013, the lifetime prevalence of sexual violence ranged from 4.4% in Cambodia to 37.6% in Eswatini (formerly known as Swaziland), exceeding 25% in most countries. Prevalence among males in these surveys ranged from 8.9% in Zimbabwe to 21.2% in Haiti (8).

In Mexico, studies have shown a concerning increase in prevalence. In 2005, 3.6% of women and 1.9% of men reported experiencing CSA (9). The 2021 National Survey on the Dynamics of Household Relationships (ENDIREH) reported that 12.6% of Mexican women aged 15 and older had experienced CSA, an increase from 9.4% in 2016 (10). The National Health and Nutrition Survey (ENSANUT) reported an overall CSA prevalence of 2.5% in adolescents (3.8% in women and 1.2% in men) from 2018–2019, which subsequently rose to 5.5% overall in 2022 (9.0% in women and 2.2% in men) (11, 12). These data illustrate that the problem is growing and disproportionately impacts women.

### 1.2 Ecological model of CSA

Child sexual abuse is a multifactorial phenomenon that necessitates a comprehensive prevention approach (13). The prevention proposal adopts the Bronfenbrenner Ecological Model, which considers the interplay of individual, family, and community factors in CSA prevention. The Bronfenbrenner Ecological Model, also known as Ecological Systems Theory (EST), provides a comprehensive conceptual framework for understanding and addressing CSA. It posits that a child’s development, socialization, and risk-protective factors are influenced by multiple, dynamic, and interacting environmental layers. The model highlights the significance of multi-level interventions for effective CSA prevention, emphasizing the need to target change across these systemic layers to achieve desired behavioral and social outcomes (14). The EST identifies four nested environmental systems that influence a child’s likelihood of experiencing or preventing harm. Comprehensive prevention requires attention to each layer. The microsystem comprises the child’s immediate, face-to-face settings and direct interactions, such as family, school, and peer groups. The mesosystem refers to the connections and coherence between two or more of the child’s microsystems. For example, the relationship or lack thereof between the home and the school. The exosystem consists of extrinsic settings that indirectly affect the child (15). The child is not an active participant here, but events in these settings -such as school administration policies, the parent’s workplace conditions, or the availability of community services and legal systems- still exert influence. The macrosystem encompasses larger factors, such as cultural norms, that normalize abuse and contribute to risk. Risk factors include inadequate child supervision (16), a lack of family support, poor parent–child relationship quality, and the absence of a nuclear structure (17).

The EST framework is designed to produce different outcomes in each system. At the microsystem level, it is expected to increase knowledge of self-esteem and body rights, as well as to increase the willingness to seek help or disclose to a trusted person. At the mesosystem/exosystem level, it is expected that children will identify harmful situations, take action to prevent violence, and strengthen linkages between schools and specialized institutions in CSA for referral. At the macrosystem level, the goals are to reduce the prevalence of CSA in the target population, to shift cultural norms by addressing sensitive topics openly, and finally, to replicate the intervention at a state or national level.

In Mexico, more than half of CSA cases involve an abuser known to the victim and/or within the family nucleus (18, 19). This proximity allows perpetrators to exploit family protective barriers and evade repercussions (20). The peak occurrence of CSA falls between the ages of six and twelve (9, 19, 21, 22). Because these are important formative years in children’s socioemotional development and self-esteem, working in preventive measures at these ages is highly recommended.

### 1.3 Educational intervention to prevent CSA

The school environment is pivotal in children’s lives at the community level, and the World Health Organization (WHO) recognizes the potential of schools and educators in violence prevention (23). School-based education has been recognized as one of the most effective strategies for CSA prevention since the 1970s, as it provides children with the necessary knowledge to protect themselves (24, 25). A comprehensive approach that integrates children, their families, and teachers is also crucial (26).

The use of technology in learning is a growing trend. Prevention programs using digital tools have proven effective in promoting body safety awareness and protective behaviors (27), in increasing knowledge about sexual abuse (19, 28, 38), as well as in increasing subject’s active intervention skills in safe immersive settings to improve prevention models (29). The Virtual Learning Environment (VLE), which is based on pedagogical models that incorporate didactic objects and unique experiences, has been shown to improve educational outcomes (30). This method positively affects knowledge retention, intensifies the user’s emotional state, and increases motivation (31). Conversely, numerous research studies suggest that Immersive Virtual Reality (IVR) holds significant potential to surpass conventional educational methods in preventing complex social issues like bullying and abuse. For instance, one specific study focusing on adolescents successfully demonstrated the efficacy of an IVR-based sexual harassment prevention program. This program was not only shown to reduce sexual victimization but also actively enhanced victims’ self-defense behaviors (29). This effectiveness is primarily attributed to IVR’s unique capacity to establish highly realistic, yet psychologically safe, simulated environments. Within these environments, users can actively rehearse and experience the consequences of their actions in an immersive manner. This facilitates the kind of deep, experiential learning that is challenging to achieve through traditional methods, such as lectures or printed materials. Furthermore, a comprehensive systematic review of digital gaming applications and Virtual Reality technologies in the management of child abuse further validates the efficacy of these innovative solutions (32). This is particularly true for interventions based on serious games in both the prevention and treatment domains. This consistent trend, supported by positive outcomes in proactive behaviors (such as victim advocacy), supports the core hypothesis: IVR, by effectively combining immersion, interactivity, and safe practice, offers a superior educational platform for fostering more effective and lasting changes in attitudes and behaviors compared to conventional approaches.

The project’s objective is to test the effectiveness of the “Pinta tu Raya ASI” preventive intervention to enhance the competencies and understanding of elementary school students in two Mexican states regarding critical CSA prevention issues.

## 2 Methods

### 2.1 Study design

**Figure 1:**
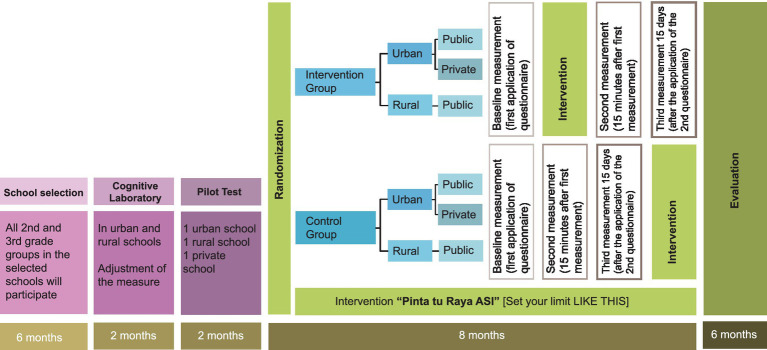
Study design.

This study is a two-arm matched cluster-randomized controlled trial that includes the psycho-pedagogical intervention “Pinta tu Raya ASI.” The intervention targets second and third-grade students (ages 7–8) in elementary schools in the Mexican states of Morelos and Nayarit. This initiative is part of a comprehensive two-year study. (Figure 1).

For school selection, a list will be requested from the Ministry of Education, focusing on municipalities with a gender violence alert designation. This designation indicates emergency governmental actions to confront and eradicate femicide and the disappearance of women. A random sampling of schools will then be conducted from this list. Prior to randomization, schools will be paired 1:1 based on specific characteristics (e.g., size, type, locality).

The required sample size for the intervention was calculated using the clustersampsi command from the STATA statistical software package, which is designed for estimating sample sizes in cluster randomized trials. The mean knowledge scores (and standard deviations) of the control and intervention groups were included, setting the number of clusters at 12 schools per arm (due to the operational capacities of the work team) (33). The intraclass correlation value was set at 0.15 (34), the power at 80%, and the statistical significance at 0.05. This resulted in a minimum sample size of 324 children per arm. Following the selection of the participating elementary schools, the randomization unit will be the schools as a whole. Specifically, all eligible children in the second and third grades within a selected school will participate in the study.

### 2.2 Intervention “Pinta tu Raya ASI” [Set your Limit LIKE THIS]

The creation of the “Pinta tu Raya ASI” psycho-pedagogical intervention involved an extensive bibliographic review of key CSA prevention themes and protective factors of the ecological model of sexual violence.

#### 2.2.1 IVR animation and workbook creation

**Figure 2:**
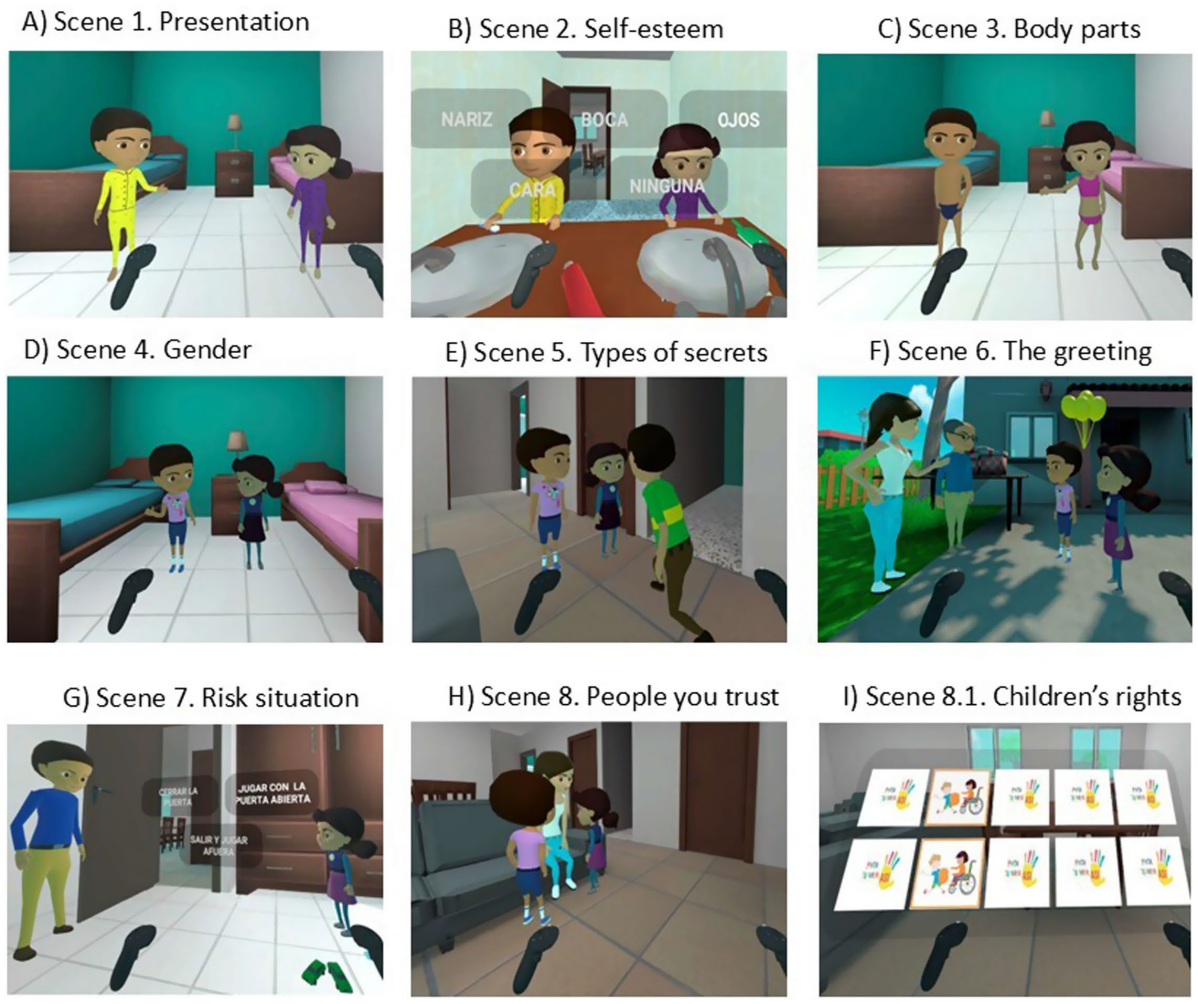
**(A)** Pablo and Sofía in pajamas welcoming the participant. **(B)** Pablo and Sofía in the bathroom, highlighting the importance of self-love, regardless of physical appearance (with labels in Spanish indicating body parts). **(C)** Pablo and Sofía explaining the private parts of the body. **(D)** Pablo and Sofía discussing gender (explaining that colors and toys are not gender-specific and that children can dress and play as they wish). **(E)** Pablo and Sofía run into their older brother, who explains the different types of secrets to them. **(F)** Pablo and Sofía leave the house and meet their mother, who introduces them to another adult. They must decide how to greet this adult. **(G)** Another adult in the garden (a family member) asks Sofia to see her room. Once in the room, Sofia must choose what to do in this situation (close the door, play with the door open, or go outside and play). **(H)** Pablo and Sofía run to see their mother to tell her about the previous situation. Information is provided about the importance of trusted people. **(I)** Pablo and Sofía explain some of the children’s rights, and a memory game appears.

A literary script and a workbook were developed, and a 3D animation was produced for the intervention’s implementation. The literary script and workbook were developed through a collaborative, multidisciplinary approach. The development team included filmmakers, theater scriptwriters, health communication specialists, and researchers with established expertise in the field of CSA. Following the creation of the educational material, it passed an initial validation phase, where it was presented to both teachers and parents during a working meeting. In the IVR, two characters, Pablo and Sofia, guide children through eight different scenes in the 3D animation (see Figure 2). The animation is estimated to last approximately 20 minutes and covers the following themes:

- Self-esteem: Understanding that they are unique and valuable, regardless of physical features.
- Body parts: Information on private and public body parts, explaining which can and cannot be seen or touched.
- Gender: Understanding that colors and toys are not gender-specific and that children can dress and play as they wish.
- Safe and Unsafe Secrets: Explaining the different types of secrets and the associated emotions.
- The Greeting: Explaining that a child is free to greet an adult in the way they prefer (kiss, hand, or hug) and is not obligated to kiss an adult (as is customary in Mexico).
- Appropriate and Inappropriate Touches: Helping children understand the difference between these types of touches.
- Child Sexual Abuse: A scenario where the child determines an action (go out, play with the door open, or play with the door closed) when the IVR character, Sofia, is left alone with an adult.
- Threats: Learning that abusers may use threats to control them and the importance of disobeying these threats and seeking out a trusted person.
- Trusted Person: Information to help children understand what a trusted person is, noting that this person may not always be a parent.
- My Rights as a Girl and a Boy: A memory game at the end of the story where children match images of the main children’s rights.

The animation will be projected using Oculus Quest IVR headsets. These headsets function autonomously, requiring no internet connection or other electronic devices, making them suitable for both urban and rural environments. The headsets allow children to feel immersed in the virtual reality experience and use their hands to answer questions or grab objects.

### 2.3 Intervention and control group

The study will encompass public, private, urban, rural, and some indigenous elementary schools. Schools will be paired 1:1 prior to randomization based on characteristics like size and locality.

The intervention group will receive an initial learning session involving the IVR animation projected with the Oculus Quest headsets. Groups of 8 to 10 students will be formed for the individual IVR projection (approximately 20-minutes). The projection will take place in a specific area of the school. Headsets will be thoroughly cleaned with a disinfectant solution before each use. Researchers and a team of psychologists will be present during the IVR session.

The control group will not receive the initial learning session but will receive it by the end of the three measurements (Figure 1).

### 2.4 Measures and questionnaire

A questionnaire was developed to assess children’s knowledge related to CSA prevention, based on a comprehensive review of international educational and preventive interventions. A group of experts selected the most important questions from a validated questionnaire (test–retest reliability *r* = 0.92, internal consistency Kuder–Richardson *r* = 0.83, and validity with the Children’s Knowledge of Abuse Questionnaire-Revised *r* = 0.76) (35). The experts retained 14 questions on topics that impact the prevention of such violence (Table 1). Following the development of the measurement instrument, two cognitive laboratories (36) will be conducted: one in an urban public elementary school and another in a rural public elementary school. These cognitive laboratories will facilitate the necessary adjustments to the questions, ensuring each one is clear and comprehensive for children. Subsequently, a pilot test will be conducted to evaluate both the IVR intervention and the knowledge questionnaire. Any necessary modifications to the instrument will be implemented considering this pilot phase. Finally, upon the implementation of the intervention, each participant will be administered the questionnaire. To ensure complete comprehension by the children, a member of the research team will read every question aloud.

**Table 1:** Questionnaire created for measuring knowledge for the prevention of child sexual abuse (CSA).

1) Imagine that someone tells you they are planning a surprise party for your teacher and insists on keeping it a secret. Wouldn’t you want to help them out and keep it under wraps?		
Yes	No	I do not Know
2) Do children have the right to go to the doctor when they are not feeling well, just like adults?		
Yes	No	I do not Know
3) If someone pinches you or squeezes your arm really hard, is it okay to say you do not like it and then walk away?		
Yes	No	I do not Know
4) If a family member or a friend wanted to touch your private parts, would you feel comfortable telling a trusted person?		
Yes	No	I do not Know
5) If someone touches your body in a way that makes you feel uncomfortable, do you think it’s your fault?		
Yes	No	I do not Know
6) Is it okay for someone who loves you to give you a hug that makes you feel good?		
Yes	No	I do not Know
7) If someone were to touch you in a way that made you feel a bit uneasy and then asked you to keep it a secret, would you keep it a secret?		
Yes	No	I do not Know
8) If something happened to you that you are embarrassed, afraid, or ashamed of, would you tell someone you trust?		
Yes	No	I do not Know
9) If someone were to touch you in a way that made you feel uncomfortable and then told you that they would hurt your family, would you tell someone you trust?		
Yes	No	I do not Know
10) Do all children have the right to live free of violence?		
Yes	No	I do not Know
11) Both boys and girls can play with any toy they want, regardless of whether it’s a “boys’ or girls’ toy.		
Yes	No	I do not Know
12) You are such a unique and special person. You are loved just as you are, and you have so much value.		
Yes	No	I do not Know
13) Should you always greet an adult person with a kiss or a hug, even if you feel uncomfortable greeting him or her?		
Yes	No	I do not Know
14) If a grown-up listens to you, believes you, makes you feel happy, takes care of you, and does not hurt you, can we say that they are a person you can trust?		
Yes	No	I do not Know

Three knowledge measurements will be taken in both groups.

- Intervention Group: Measurement will be taken prior to the intervention (baseline), immediately after the IVR session, and 15 days later.
- Control Group: Measurement will be conducted at baseline, followed by two follow-up phases: 20 min after the initial measurement and 15 days later (Figure 1).

### 2.5 Ethical aspects

The study protocol was approved by the Research Ethics and Biosafety Committees of the National Institute of Public Health (registration number CI: 1713 V47). Informed consent letters were prepared for the parents/legal guardians of the minor participants, and informed assent letters were prepared for the children. Participation will be limited to children who will have both documents signed.

Due to the sensitivity of working with CSA, researchers will first approach school authorities to explain the project’s objective, followed by contact with parents and caregivers to extend invitations. The approach with the children will be carried out by personnel sensitized to CSA and experienced in fieldwork with children. The researchers will explain that the project aims to teach children key information via the IVR headsets to help them take care of their bodies and avoid harm.

Researchers recognize that some children may experience discomfort or anxiety when wearing the IVR headsets. Therefore, the researchers will first ascertain the child’s willingness to participate, even if assent is signed. Second, following the placement of the IVR headsets, the psychology staff and researchers will observe the children’s body language to identify signs of anxiety or discomfort. If a child indicates they no longer wish to participate, the headsets will be removed, and their information will be excluded from the study.

To ensure optimal outcomes, the intervention includes a team of specialized psychologists, trained to work with CSA cases, as the intervention may detect such cases. To this end, strategic partnerships will be stablished with specialized CSA intervention institutions. This collaboration is designed to facilitate appropriate referral of identified abused children to the *Sistema Nacional para el Desarrollo Integral de la Familia* (National System for the Integral Family Development, SNDIF in Spanish). The SNDIF is a decentralized Mexican public agency responsible for providing social assistance and safeguarding the rights of vulnerable populations, including children. Based on the empirical findings of the current intervention, a second phase will focus on developing and implementing targeted training programs. These programs will be delivered to teaching staff and parents to strengthen their capabilities in the detection and appropriate referral of CSA cases to the relevant institutional authorities.

### 2.6 Data management

The information of the questionnaires will be stored in a password-protected Excel file, and only the research team will have access to this document. To ensure the confidentiality and security of student information, children will not be identified by name. Instead, an alphanumeric code will be created based on their state, school, grade, and classroom.

### 2.7 Statistical analysis

A descriptive analysis of the baseline characteristics (schools and students) will be conducted. Continuous variables will be reported using the mean and standard deviation. Categorical variables will be presented as frequencies and percentages. We will employ a Propensity Scores matching strategy to create comparable intervention and control groups based on observed baseline covariates.

Following the matching, covariate balance will be assessed using the t-test for continuous variables and the chi-square test for categorical variables. This post-matching evaluation is essential for validating the matching procedure and confirming the similarity of initial group characteristics. The impact of the intervention will be evaluated using a mixed-effects generalized least squares model. This model is specifically selected to address the hierarchical data structure (students nested within matched schools) and the study’s inherent cluster randomized design. The model will include fixed effects at the matched pair level and random effects at the student level. The analysis will also explore subgroup effects by assessing how the intervention’s impact varies across key demographic variables, including school location (rural vs. urban) and school type (public vs. private).

If the percentage of missing data is less than 5%, imputation is not necessary; biases are minimal if the data are mostly Missing Completely at Random (MCAR). The analysis can be performed with complete cases (listwise deletion). If the percentage of missing data is greater than 5%, missing values will be handled primarily by multiple imputation using chained equations (MICE) that account for the hierarchical data structure (school and individual levels). Sensitivity analyses (complete-case, inverse probability weighting, and, if needed, MNAR-based models) will be performed. The proportion of missing data per variable and cluster will be reported. The primary outcome will be analyzed at a two-sided *α* = 0.05. For multiple comparisons, the false discovery rate (FDR, Benjamini–Hochberg) or Bonferroni corrections will be applied as appropriate. All estimates will be presented with 95% confidence intervals and both raw and adjusted *p*-values The whole analysis will be done using Stata 17.0 software (College Station, TX).

## 3 Discussion

Research indicates that educational interventions successfully enhance individuals’ knowledge of sexuality. However, determining the extent to which these interventions actually prevent CSA remains challenging due to the paucity of empirical data on program effectiveness in real-world settings (22, 37). The advent of the VLE has enabled the rigorous evaluation of digital tools in CSA prevention. Studies utilizing VLEs have demonstrated a significant enhancement of knowledge and key conceptual understanding related to CSA prevention in experimental groups (38). Therefore, there is a compelling need to strengthen children’s learning through an educational intervention based on IVR. Immersive Virtual Reality has been shown to have positive effects on knowledge retention, motivation, and learning outcomes (39).

“Pinta tu Raya ASI” incorporates didactic objects to provide users with unique, practical experiences that are not feasible in the physical world seeking an increase in knowledge of key topics. The system effectively represents realistic scenarios while eliminating physical risks and allowing for the precise regulation of conditions, ensuring suitable and safe presentation for the training group. The intervention is innovative, captivating, and highly relevant for children in the second and third grades, as it utilizes cutting-edge technological tools. It is evident that learning reinforced through IVR will play a pivotal role in strengthening children’s understanding of related textbook topics. Furthermore, implementing the intervention in the second and third grades is imperative because these children are within the age range associated with the highest risk of CSA victimization (9, 21, 22). It is important to acknowledge, however, the potential for expanding the intervention’s scope beyond elementary school children to encompass a triad of student-teacher-family training. This comprehensive approach will be considered during later developmental stages of our project to maximize CSA prevention efforts.

### 3.1 Strengths and limitations

Among the main strengths of this project we can outstand this is the first immersive virtual reality educational intervention to focus on CSA prevention with children in Mexico, besides it will be the first one to be evaluated. Furthermore, this represents a significant innovation in how we address the issue of CSA in our country and in Latin cultures. We can also highlight the importance of creating safe environments for working on this sensitive topic with children in a playful way, and the advantage of involving schools (administrators and teachers) and parents in CSA prevention. About possible limitations, probably the most important one is the future scalability of this project due to the initial capital expenditure required for purchasing the IVR headsets. However, once the hardware is acquired, replication costs will be minimal, as the intervention program itself will be freely accessible and user-friendly for installation. Another potential challenge during the intervention’s initiation is family reluctance to engage, given the sensitive nature and prevailing cultural taboo surrounding CSA in our society. To mitigate this, we have planned a sensitization initiative targeting school management, teaching staff, and families, aimed at fostering an optimal and positive response rate. We also anticipate the potential for measurement bias stemming from pre-exposure to violence or abuse prevention content delivered by teachers. To address this, a thorough review will be conducted with school administration prior to school selection to ensure that participating children have not recently received comparable information. An additional challenge is the risk of technical failures with the IVR headsets during the intervention. This issue will be addressed proactively by conducting a pilot study to ensure the proper functionality and stability of all technological aspects of the program. Finally, a key limitation of the study’s external validity is the selection of schools from only two Mexican states, which confines the direct generalizability of the results to these specific regions. However, should the intervention prove effective, we anticipate that the results will inform and facilitate the replication and subsequent scaling of the program across other states throughout the country.

## 4 Conclusion

The “Pinta tu Raya ASI” program is a novel, comprehensive, and educational intervention designed to prevent CSA among elementary school children in Mexico through IVR technology. The program is expected to significantly enhance students’ learning and skills related to self-esteem, body safety, and rights, ultimately addressing child violence in Mexico.

The intervention’s primary strength is its innovation and feasibility, as it requires only IVR headsets that can be used universally, irrespective of internet access. While the initial headset purchase is a capital expenditure, the program will be freely accessible and user-friendly for replication.

Although the initial study is limited to two Mexican states, successful results are intended to lead to replication across the country. The project also plans to expand its scope later to a comprehensive student-teacher-family triad training approach for broader CSA prevention. This study is anticipated to serve as a model for other childhood violence prevention interventions in diverse cultural, social, and economic contexts.
